# Legal document similarity: a multi-criteria decision-making perspective

**DOI:** 10.7717/peerj-cs.262

**Published:** 2020-03-23

**Authors:** Rupali S. Wagh, Deepa Anand

**Affiliations:** 1 Department of Computer Science, JAIN Deemed to be University, Bangalore, Karnataka, India; 2 Department of Information Science and Engineering, CMR Institute of Technology, Bangalore, Karnataka, India

**Keywords:** Legal Information Retrieval, Concept Based Similarity, Multi-Dimensional Similarity, OWA, Concept interaction graph

## Abstract

The vast volume of documents available in legal databases demands effective information retrieval approaches which take into consideration the intricacies of the legal domain. Relevant document retrieval is the backbone of the legal domain. The concept of relevance in the legal domain is very complex and multi-faceted. In this work, we propose a novel approach of concept based similarity estimation among court judgments. We use a graph-based method, to identify prominent concepts present in a judgment and extract sentences representative of these concepts. The sentences and concepts so mined are used to express/visualize likeness among concepts between a pair of documents from different perspectives. We also propose to aggregate the different levels of matching so obtained into one measure quantifying the level of similarity between a judgment pair. We employ the ordered weighted average (OWA) family of aggregation operators for obtaining the similarity value. The experimental results suggest that the proposed approach of concept based similarity is effective in the extraction of relevant legal documents and performs better than other competing techniques. Additionally, the proposed two-level abstraction of similarity enables informative visualization for deeper insights into case relevance.

## Introduction

Easy availability of legal information resources through online legal databases has provided much-required acceleration to the research in the domain of legal information retrieval (LIR). LIR aims at retrieving legal information objects relevant to a user’s query. Legal information objects are various documents like court transcripts, verdicts, legislation documents, and judgments that are generated during the course of a legal process. These documents are primary resources for the interpretations of the law of any judiciary and hence are required by a legal professional for decision making as well as argumentation. Specific characteristics of legal documents like document size, document internal structure, temporal properties, specific legal terminology, polysemy, and heterogeneity make LIR different extremely complex as compared to other domains. Since every legal document presents one or more legal issue, the legal domain demands context based document retrieval than just data based retrieval. Contextualization of a legal issue is a non-trivial task due to the inherent complexities of this domain. Additionally, the concept of “match” or “relevance” is multi-dimensional in the legal domain (Van Opijnen, 2012). LIR is thus a very challenging research field as the domain necessitates for very generic to a very specific abstraction of a legal document at the same time. Retrieving relevant legal document from a huge collection of resources requires a deep understanding of the notion of relevance in this domain and intelligent methods for identification and representation of legal concepts for establishing relevance.

Finding similarity among legal documents, specifically among court judgments is one of the most studied problems under LIR. Methods and techniques used in LIR originate from confluence of four major technologies: namely Artificial Intelligence (AI), Network Analysis, Machine Learning and NLP (Bench-Capon et al., 2012). Legal knowledge is very complex and is available in various documents written in natural languages. Ontology, a branch of AI, is widely used to facilitate effective knowledge management in the legal domain (Saravanan, Ravindran & Raman, 2009). Knowledge engineering using semantic web and ontology for specific sub-domains of law is practiced popularly (Casanovas et al., 2016) due to the ease of modeling legal actors, agents, and relationships using these technologies. With the advents in other technological domains, legal ontological solutions are also upgraded to incorporate more scalable, re-usable, context-aware and user-centered approaches in the existing framework. Citations or bibliographical relevance in the legal domain is extremely important for understanding the interpretations and applications of law and a network is the most obvious representation of data for legal citation analysis. Thus, citation network analysis explicably remains one of the very popular techniques in LIR. Earlier approaches predominantly use network degree statistics and structural properties for extraction of relevant documents in the legal domain (Van Opijnen, 2012; Koniaris, Anagnostopoulos & Vassiliou, 2017). Approaches which use centrality and between-ness of a node in a case citation network (Wagh & Anand, 2017) to find similarity among Indian court judgments are proposed. But, with the recent advancements in deep learning based graph embedding models (Cui et al., 2018), graph and all its components can be represented as dense feature vectors enabling exploration of newer models in network analysis for LIR. (Sugathadasa et al., 2018) use node embeddings obtained using node2vec algorithm (Goyal & Ferrara, 2018; Grover & Leskovec, 2016) for case citation data for finding similar legal documents. Analysis of case citation data using machine learning methods to estimate similarity among cases has also been experimented in the past. Coupling of bibliographic information with text in the paragraph of judgments (Kumar et al., 2013) for estimation of similarity between two judgments is proposed. Exploring relatedness among cases by finding common citations is proposed (Nair & Wagh, 2018) where authors present application of association rule mining to estimate similarity value. While citation based similarity among court cases is undoubtedly of very high significance in legal domain, the case citations graphs are generally very sparse (Mandal et al., 2017a; Mandal et al., 2017b). Moreover, semantic relationships among the case judgments and their interpretation are implicitly available as text within a judgment document. Natural language processing (NLP), along with machine learning methods are used to establish semantic relevance of textual contents present in the documents (Branting, 2017). Until recently, Vector Space Model and Latent Semantic Indexing, LSI with its variants were used largely for semantic representation of text. With the emergence of word/document embeddings, information retrieval is now shifted to neural information retrieval (Onal et al., 2018). Dense vector representations of word and document obtained using deep learning based models are used as input for machine learning algorithms. The strength of these models lies in capturing the semantics of text and thereby recognizing document similarities without exact word-match. Many studies highlight the effectiveness of neural embedding for text Mandal et al. (2017a), Mandal et al. (2017b) and Vo, Privault & Guillot (2017) for Legal Information retrieval.

Finding relevant precedents (judgments) is one of the most widely studied problems in LIR. A court judgment is a complex document with various sections describing the application of law to the legal issues discussed during the case proceedings. There is a general agreement on the need for concept-based document retrieval in legal domain, and the approaches for LIR largely focus on obtaining a single representation of document covering all legal concepts present in the document which results in single similarity value. One of the major limitations of these approaches is the inability to provide interpretations of relevance for in-depth understanding. While a single numeric value for measuring relevance is undoubtedly of very high significance in information retrieval, user satisfaction in an IR system also depends on intuitively informative results provided by the system. There are studies (Koniaris, Anagnostopoulos & Vassiliou, 2017) emphasizing on the need for going beyond a single homogeneous similarity value for more effective legal information retrieval. In this proposed work, we present the legal document similarity estimation as a multi-criteria decision-making (MCDM) problem. We specifically focus on the problem of finding the similarity among court judgments for the Indian Supreme Court judgment corpus. We extract prominent concepts which are considered as criteria and extract representative sentences for each of the criteria. Using these sentences, we then generate a concept similarity matrix for the concepts extracted from the documents. Every value in the similarity matrix represents weight for the criterion and final similarity value is calculated using the ordered weighted average (OWA) operator. Thus, the approach provides two abstractions of relevance between a judgment pair: (1) At the concept level as a matrix of similarity values; (2) at the document level as single similarity value obtained by aggregating concept level similarity. Experimental results demonstrate the effectiveness of our proposed approach for the extraction of relevant judgments. In addition to the enhanced performance of relevant judgment retrieval, this approach enables informative visualization of results that provide deeper insights into the relevance obtained.

The remainder of the paper is organized as follows; the next section, ‘Materials and Methods’, elaborates the steps of the proposed approach in detail. The section ‘Experimental Evaluation’ discusses the experimental set-up and provides the details on the data set and implementation framework used in the proposed work. We present results and discussion on obtained results in the ‘Results and Discussion’ section where we compare the results with existing work in LIR for Indian Legal System. We further highlight the effectiveness of our work. We conclude with a note on the future direction for the proposed work in the ‘Conclusion’ section.

## Materials & Methods

Semantic matching of documents is the most fundamental activity of LIR. Generically, textual units of different granularity viz. words, phrases, sentences, paragraphs and even complete documents are used for establishing semantic relevance between user’s query and documents. Embeddings are obtained by considering word neighborhood as the context, and hence capture the semantics of text even without exact word match. These methods are very effective and popular for all NLP tasks across the domains (Onal et al., 2018). One of the limitations of deep learning based vector embedding as highlighted in (Moody, 2016) is the inability to provide interpretative insights. Judgment documents are complex and lengthy. The estimation of similarities among long documents requires a different approach as the similarity has to be modeled as a function of the concepts present in the documents (Liu et al., 2018). Moreover, since the concepts may be scattered throughout the body of the text in a document, a well-defined approach for identification of concepts is required. In this paper, we propose a three-step approach for finding concept based similarity among court judgments: (i) Identification of main concepts/topics of the document (ii) Extraction of the text under every concept (iii) Similarity calculation using suitable measure. These steps are explained in detail in the following sub-sections.

### Identification of basic concept words

Natural Language Processing (NLP) offers a wide range of methods and approaches for the identification of topics from a document. Traditional TF-IDF based vector space model and Latent Dirichlet Allocation (LDA) use the distribution of words (Moody, 2016) in the document to extract topics. These methods do not consider word neighborhood and are based on exact word match. Graph-based extraction of topics is another popular approach (Ying et al., 2017 and Sayyadi & Raschid, 2013) for identifying the broad themes in documents. These methods are based on establishing a relationship between words/concepts using estimates such as co-occurrence, semantic similarity, etc. for extraction of prominent topics in a document. Variants of the above two approaches are popularly used for topic identification and available as off-the-shelf tools for identifying prominent words in the document.

We propose employing a variation of the graph-based method for identifying topics and utilizing it to obtain important segments of the judgment. Let $\mathfrak{L}= \left\{ {\mathfrak{L}}_{1},{\mathfrak{L}}_{2}\ldots ,{\mathfrak{L}}_{n} \right\} $ be the set of ‘n’ legal judgments in the corpus. Let *n*(𝔏_*i*_) be the set of sentences in the legal document 𝔏_*i*_ and let 𝔏_*ij*_ be the ‘j’th sentence of the ‘i’th legal judgment document. As the first step in the pre-processing of documents, we construct the base concept words as the nouns present in sentences in the judgment. Liu et al. (2018) propose extraction of keywords as basic concepts where authors demonstrate similarity estimation for news reports using concept interaction graph. Specific and distinctive characteristics of legal documents require a domain-specific approach for the extraction of concepts from the documents. While a person’s name may have a lot of relevance in a news report, it just represents a party (respondent or appellant) or a participant in the case and does not actually contribute to any legal concept present in the judgment. Therefore, we ignore references to specific people, place etc. which appear as proper nouns from the input in the document, and we define base word concept set of the ‘j’th sentence in the ‘i’th document, $\mathfrak{B} \left( {\mathfrak{L}}_{ij} \right) $, as:

(1) \begin{eqnarray*}\mathfrak{B} \left( {\mathfrak{L}}_{ij} \right) = \left\{ x\in {\mathfrak{L}}_{ij} \right\vert pos \left( x \right) ={}^{{}^{{^{\prime}}}}CommonNou{n}^{{}^{{^{\prime}}}}and x\in \Im ({\mathfrak{L}}_{ij})\end{eqnarray*}

Here $pos \left( x \right) $stands for part of speech of the word x and ℑ(𝔏_*ij*_) represents the important words in the sentence 𝔏_*ij*_. We consider a common noun appearing in the sentences as concepts and construct a concept interaction graph using concept co-occurrences. However, we are selective about the nouns appearing in the concept graph and only allow important nouns to represent the document fragments. TF-IDF, term frequency-inverse document frequency method is the most fundamental weighing scheme used in an information retrieval system. TF-IDF computes weight for a term in a document collection by assessing its local relevance using term frequency within the document (TF) and global relevance by computing inverse document frequency for the entire document collection (Ramos, 2003). To assess this importance of the nouns we use TF-IDF model constructed for individual judgment by considering every sentence in a judgment as a separate document. The judgment, therefore, can be deemed to be a collection of documents (${\mathfrak{L}}_{ij},j\epsilon 1\ldots n \left( {\mathfrak{L}}_{i} \right) $). Therefore $\Im \left( {\mathfrak{L}}_{ij} \right) $ can be determined as:

(2) \begin{eqnarray*}\Im \left( {\mathfrak{L}}_{ij} \right) = \left\{ x\in {\mathfrak{L}}_{ij} \right\vert tf \left( x,{\mathfrak{L}}_{ij} \right) \times idf \left( x,{\mathfrak{L}}_{i} \right) \gt \mathit{mean}_{\mathit{k\epsilon }[1,n \left( {\mathfrak{L}}_{i} \right) ]}(tf \left( x,{\mathfrak{L}}_{ik} \right) \times idf \left( x,{\mathfrak{L}}_{i} \right) )\end{eqnarray*}

where *tf*(*x*, 𝔏_*ij*_) is the term frequency of the word ‘x’ in the sentence 𝔏_*ij*_, $idf \left( x,{\mathfrak{L}}_{i} \right) $ measures the uniqueness of ‘x’ across the document 𝔏_*i*_ and the words having TF-IDF above the mean TF-IDF score over the document are considered important.

### Identification of main concepts/topics of the document

**Figure 1:**
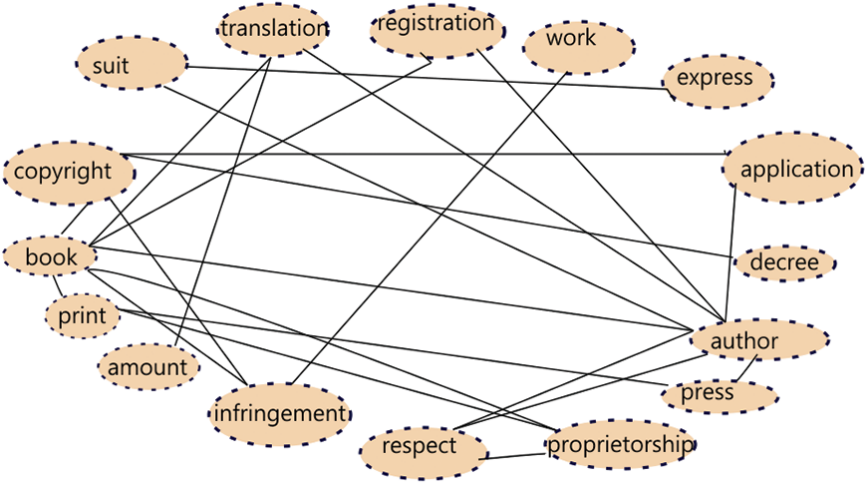
Sample Concept graph for a judgment document fragment.

Detection of related words in the judgment document is an important step and this is assessed based on the proximity of the base concept words. A concept graph 𝔊_*i*_ = (𝔙_*i*_, 𝔈_*i*_) of a legal document 𝔏_*i*_,  is constructed using the base concept words s.t. ${\mathfrak{V}}_{i}={\bigcup }_{j\in \left[ 1,n \left( {\mathfrak{L}}_{i} \right) \right] }\mathfrak{B} \left( {\mathfrak{L}}_{ij} \right) $ and ${\mathfrak{E}}_{i}= \left\{ \left( x,y \right) {|}co-occurrence \left( s,y \right) \gt 3 \right\} $. The set of vertices 𝔙_*i*_ is the set of all base concept words across all sentences in the document and two concept words nodes in the graph have an edge between them if their co-occurrence count is above 3 i.e., they appear together in at least three of the sentences. We use the count of co-occurrences as the strength of association between two concepts words. Less than 3 co-occurrences of concept words may represent mere coincidence and hence we do not deem such associations as strong enough for addition of edge in the graph. Figure 1 shows a concept graph constructed from a document fragment.

**Figure 2:**
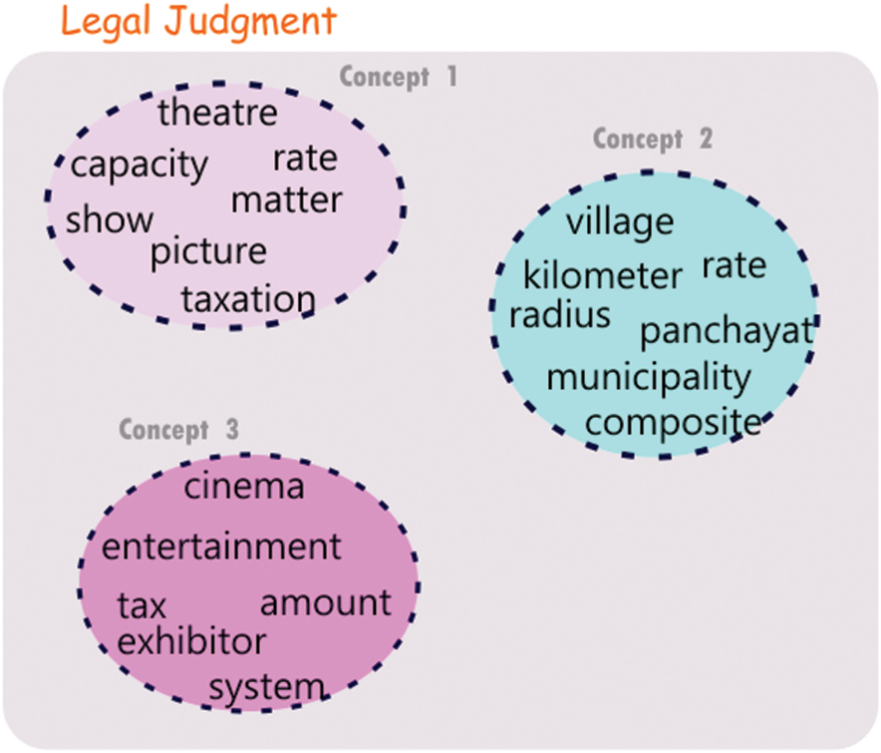
Communities derived from a judgment document.

To discover important topics in the document we employ Louvain Modularity community detection algorithm (De Meo, 2011). The algorithm tries to locate communities by trying to maximize the modularity i.e., the ratio of the density of edges inside each community to the density of edges to nodes outside the community. The algorithm runs iteratively by first identifying small communities with high modularity and then proceeds to enlarge the communities by grouping them to achieve the maximum increase in modularity. We use the best partition method in the python networkx module, for detecting concepts in the document (Community detection for Networkx’s Documentation, 2010). Figure 2 shows an example of communities so evolved for a pair of judgments. Let *m*_*i*_ be the number of communities learnt for the document 𝔏_*i*_ and let the communities so detected be *ℭ*_*i*1_, *ℭ*_*i*1_, …, *ℭ*_*im*_*i*__. Each community thus identified is considered as a prominent concept which is represented by a set of words that formed the nodes in the initial concept graph.

### Representative sentence selection and similarity estimation

Once the main concepts, represented as word communities, in the document are identified, for each concept the top five, most representative sentences for that concept are selected. TF-IDF scoring is used for this purpose. Each concept *ℭ*_*ij*_, is a collection of words and can be considered as a document, similar to how each sentence in 𝔏_*i*_ is considered a document (Eq. (2)). Cosine similarity is computed between vectors representing each sentence in the judgment with the vector representing the concept. The five most similar sentences to the concept *ℭ*_*ij*_ are chosen as sentences representing that concept in the judgment 𝔏_*i*_.

Our aim is to construct a vector representation of each concept occurring in a legal judgment which can capture the degree of occurrence of various ideas. These vector representations of concepts can ease the computation of similarity between two judgment documents. Let ${\mathfrak{S}}_{ij}^{k}$ be the kth representative sentence for the ‘j’th concept in ‘i’th legal document. The vector representation of each concept can be derived in various ways, the simplest one, being averaging the TF-IDF scores of ${\mathfrak{S}}_{ij}^{k},k\in [1,5]$ i.e., averaging the TF-IDF scores for all sentences representative of a concept. However, we leverage on recent advances in neural networks, to take advantage of the potential knowledge captured by word embeddings. Word embeddings convert each word into a multi-dimensional vector of features in such a way that the vector representation of related words has high similarity. Word embeddings are often trained on huge real-world datasets and thus are able to capture the semantics very effectively. In addition, word embeddings obtained through popular methods like word2vec (Mikolov et al., 2013) have the property of additive compositionality i.e., the ability of the sum of word vectors of words composing a sentence/paragraph to preserve the semantics contained therein. Studies indicate that a word representation obtained using a combination of neural embeddings and TF-IDF provides is more effective (Lau & Baldwin, 2016) than the just the vector representations in many NLP tasks. Hence, we use IDF value for every word as weight applied to the vector of the word obtained using word2vec. We compute the vector 𝔚_*ij*_ corresponding to each concept *ℭ*_*ij*_ using two methods namely word2vec and IDF weighted word2vec and resultant vectors for these methods are computed using Eqs. (3) and (4) respectively.

(3) \begin{eqnarray*}{\mathfrak{W}}_{ij}=\sum _{k=1}^{5}\sum _{x\in {\mathfrak{S}}_{ij}^{k}}word2vec(x)\end{eqnarray*}

and

(4) \begin{eqnarray*}{\mathfrak{W}}_{ij}=\sum _{k=1}^{5}\sum _{x\in {\mathfrak{S}}_{ij}^{k}}word2vec \left( x \right) \ast IDF(x)\end{eqnarray*}

Here the summation involves vector addition of the word vectors of words belonging to each of the five representative sentences for the concept *ℭ*_*ij*_.

The above-computed vector representation for each concept present in the judgment is finally used to compute the similarity between judgment documents. The notion of similarity among documents, sometimes, may not be sufficiently captured by single similarity value. Two documents may be similar to each other to different degrees when observed using different viewpoints. As an example, two legal documents may be similar because of commonalities in the case history but may be different in the way the cases were argued. On the other hand, two other legal documents may have nothing common in terms of the facts of the case but both may be overturning the judgment made by a lower court. To that extent, the two cases can be considered similar. When similarity computation is employed for judging the closeness of two documents, the context of the search may be unknown. In such cases estimating similarities using different notions and visualizing the same may be more helpful to the user than obtaining a single similarity score.

**Figure 3:**
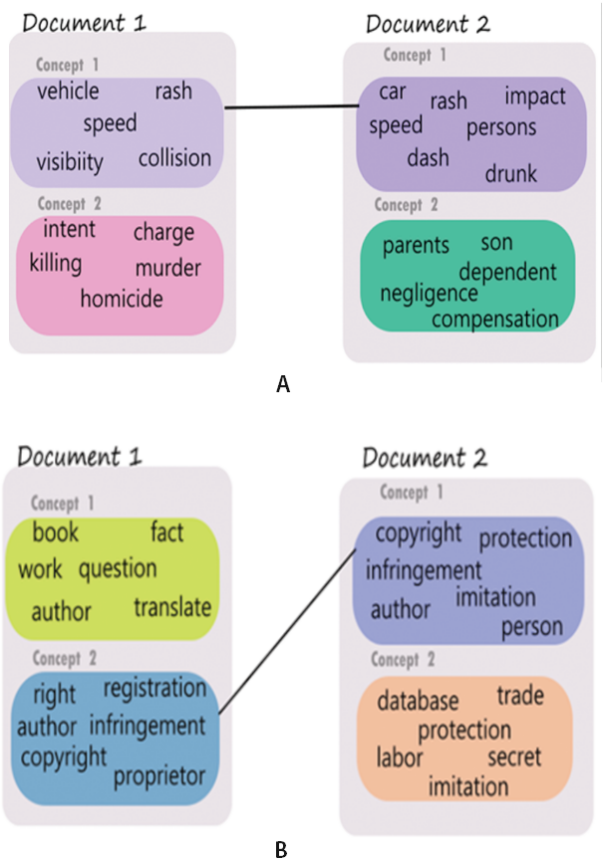
Concept based similarity—examples showing a substantial match through one concept but negligible match in another. (A) Concepts derived from accident related cases. (B) Concepts derived from copyright related cases.

The ability to derive multiple vector representations for various concepts contained in a legal document in this proposed approach could aid in finding different levels of similarity between a pair of legal documents. Let 𝔏_*a*_ and 𝔏_*b*_ be two legal judgments consisting of 𝔫_*a*_ and 𝔫_*b*_ concepts respectively. We compute the similarity between each pair of concepts in 𝔏_*a*_ and 𝔏_*b*_. Let *sim*(*ℭ*_*ai*_, *ℭ*_*bj*_) be the similarity between the ‘i’th and the ‘j’th concepts of the documents 𝔏_*a*_ and 𝔏_*b*_, respectively. In this way, we obtain 𝔫_*a*_ × 𝔫_*b*_ similarity values. We use these similarity values to establish links between concepts across the two documents. For the proposed approach, we only allow each concept to participate in a maximum of one link. Modifications to this restriction are always possible and could possibly result in different similarity links and visualization result. The concepts in the two documents having the highest similarity value are linked using a similarity link. The documents which have already been linked are removed from further linking. This process is repeated taking the next two concepts one from each of the documents which are most similar and so on. The linking of highest matching concepts between a pair of judgments would be referred to as concept matches and an example of such a concept match is illustrated in Fig. 3. It is to be noted that in Figs. 2 and 3 only the concept words are shown (rather than the representative sentences) for ease of understanding. The strength of the lines connecting various concepts across the judgments are indicators of the level of match between the concepts. We present the following two examples to support the above explanation and to demonstrate how the proposed method is able to facilitate multi-level concept matching and visualization.

Example 1: A judgment pair discusses accident as a common theme but the facts in individual case result in multiple communities. Whereas there is a high similarity match in the discussion about the accident incident itself (Concept 1 in both judgments - shown as a bold link between the two), there is little match in Concept 2 of the pair, the first talks about charges of homicide whereas the second talks about negotiating the amount of compensation for the dependents of the deceased.

Example 2: A judgment pair with discussion on intellectual property rights (IPR) and copyright. The two cases present different dimensions of IPR. Case 1 discusses IPR with respect to copyright of literary work whereas case 2 discusses copyright on customer database as a business secret. Concept 2 and Concept 1 for the pair have a high likeness since these statements talk about property rights and infringement in general; the other two concepts in the judgments discuss copyright w.r.t. books while in the second judgment the unmatched concept discusses copyright on a database, trade, etc.

Example visualization for the above two situations is shown in Fig. 3A and Fig. 3B respectively. It is to be noted that the colors of the concept nodes are representative of the degree of closeness of the concepts.

The different levels of similarity so obtained can also be aggregated to compute a single similarity value which could be useful for finding all relevant documents to a given judgment. Given the various similarity values viewed from different perspectives between two judgments, we employ the Ordered Weight Averaging operator for aggregating the various similarity values into one. OWA is a family of aggregation operators introduced by Yager (2003) has a special application for multi-attribute decision-making problem especially in the presence of fuzzy data and allows for the incorporation of linguistic criteria for aggregation. Specifically, if there are items in a domain that need to be evaluated according to ‘p’ criteria $ \left( {\mathfrak{T}}_{1},{\mathfrak{T}}_{2},\ldots ,{\mathfrak{T}}_{p} \right) $ s.t. 𝔗_*j*_(*item*) is the extent to which ‘item’ satisfies the ‘j’th criterion, then it is possible to use the family of OWA aggregation operators to evaluate the degree to which ‘item’ satisfies “some criteria”, “all criteria”, “most criteria” etc. In the case of similarity estimation in the present case, we can consider the pair of judgments to be the item and the various possible criteria could be: degree of Match in facts of the case, degree of match in case citations, degree of Match in the defense counsel’s argument, etc. In this case, the pair of judgments would be evaluated according to each of the criteria and according to our choice of linguistic aggregation needed i.e., most, some, etc, the overall similarity can be computed. It is to be noted here that the set of criteria for legal judgments is not fixed and is determined for each document pair based on the concepts derived in each document.

The OWA operator (Yager, 2003) is defined as

Definition: OWA Operator A function *f*:*R*^*n*^ → *R* is called on Ordered Weighted Averaging (OWA) operator of dimension ‘n’ if it has an associated weighting vector W of dimension ‘n’ such that:

\begin{eqnarray*}(1)\sum _{i=1}^{n}{W}_{i}& =1 \end{eqnarray*}

\begin{eqnarray*}(2){W}_{i}\in \left[ 0,1 \right] \forall i& =1,2\cdots \,,n \end{eqnarray*}

Where, F is defined as $F \left( {x}_{1},{x}_{2},\ldots ,{x}_{n} \right) ={\mathop{\sum }\nolimits }_{i=1}^{n}{W}_{i}{y}_{i}$, where *y*_*i*_ is the ‘i’th largest value in the set of elements {*x*_1_, *x*_2_, …, *x*_*n*_}.

OWA can be used to emulate different aggregation operators such as max, min, average, etc, by adjusting the weights *W*_*i*_, ∀*i* = 1, 2⋯, *n*, suitably. These linguist operators fall in between the extremes of “at least one” to “all”.

In the current work, we propose to use the “most” aggregation operator. In this paper, we just outline the method of arriving at the weights for the OWA operator and do not discuss the reasoning behind it. An in-depth presentation of the OWA operators is presented in (Carlsson & Fullér, 1996). If there are ‘p’ criteria for evaluating the similarity between a pair of documents (i.e., p concepts matches between a pair of documents), then we define an operator Q_most_, corresponding to the linguistic quantifier “most” as ${Q}_{\text{most}} \left( \mathrm{x} \right) ={\mathrm{x}}^{2}$. Then the weights for the OWA_most_ operator can be determined by the formula (Carlsson & Fullér, 1996) as:

(5) \begin{eqnarray*}\mathrm{W} \left( \mathrm{i} \right) = \mathrm{Q} \left( \frac{\mathrm{i}}{\mathrm{p}} \right) -\mathrm{Q} \left( \frac{\mathrm{i}-1}{\mathrm{p}} \right) \end{eqnarray*}

**Figure 4:**
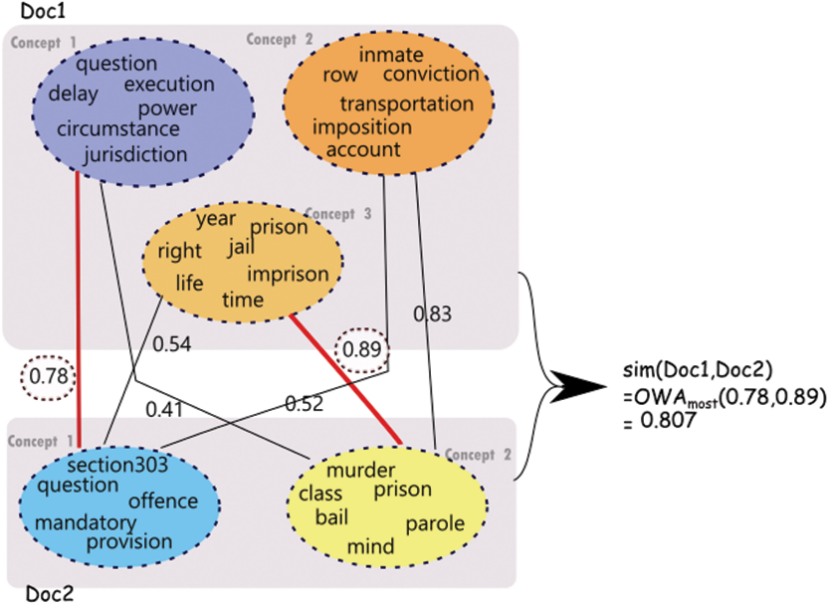
Similarity computation using communities derived for a pair of judgments. Note that the concept node colors reflect the similarity between concepts.

Figure 4 depicts similarity estimation using OWA as described above for a sample pair of judgments. As shown in the figure, for the first judgment (Doc1), three concepts are identified which are represented using three corresponding set of words. For the second judgment (Doc2), two concepts are identified. The computation of similarity depicted in the figure is performed on the sentences representative of these concepts as explained above.

**Table 1:** Extraction of concepts and representative sentences—sample results.

Case	Concept	Most Representative Sentences for the concept	Weight
Case 1	‘author’, ‘time’, ‘detent’, ‘order’, ‘ground’	‘When the Act contemplates the furnishing of grounds of detention ordinarily within five days of the order of detention the intention is clear that the statements and documents which are referred to in the grounds of detention and which are required by the detenu and are expected to be in possession of the detaining authority should be furnished with reasonable expedition.	0.583
		“That was obviously necessary because the information leading to the order of detention was laid by the Customs authorities.The grounds of detention were also served on her on the same day.It was received by the Home Department of the Delhi Administration on January 11, 1980 but was actually placed before the Administrator on January 19, 1980 when the detaining authority confirmed the order of detention.	0.447
		The authorities who laid the information before the detaining authority and who were primarily concerned in the matter were the Customs authorities via the Director of Revenue Intelligence.	0.335
	‘detenu’, ‘represent’, ‘hear’, ‘delay’, ‘right’	‘There was inexcusable delay in enabling the detenu to make a representation and indisposing of the representation.In Sukul\’s case (supra) the Court also made certain pertinent observations (at pages 231–232):\n”No definite time can be laid down within which a representation of a detenu should be dealt with save and except that it is a constitutional right of a detenu to have his representation considered as expeditiously as possible.(supra) the detenu made his representation on 4th and 6th of March 1978, the Advisory Board gave a hearing on 13th March and the detaining authority rejected the representation on 18th March.	0.516
		The rejection of the representation was communicated to the detenu on January 17, 1980.	0.462
		We have ourselves examined the records and we find that though the Administrator considered the representation of the detenu after the hearing by the Board, the Administrator was entirely uninfluenced by the hearing before the Board.	0.374
Case2	‘order’, ‘detent’, ‘opinion’, ’ground	‘Under section 7 of the Act grounds of order of detention are to be disclosed to the persons affected by the order not later than 5 days from the date of detention and the Act further requires to afford the person affected by the order the earliest opportunity of making a representation against the order to the appropriate Government.On 6 January, 1969 the Governor was pleased to confirm the order of detention after the Advisory Board had given opinion that there was sufficient cause for detention of the petitioner.	0.540
		By an order dated 26 August, 1969 the Governor was pleased to confirm the order of detention of the petitioner.(2) the opinion of this Court in the case of Sk. Section I I of the Act states that the Government may confirm the detention order if the Advisory Board gives an opinion to that effect.’	0.516
	‘detenu’, ‘releas’, ‘matter’, ’section7′, ‘right’, ‘action’	‘If thereafter the Advisory Board will express an opinion in favour of release of the detenu the Government will release the detenu.If the Advisory Board will express any opinion against the release of the detenu the Government may still exercise the power to release the detenu.	0.527
		If the appropriate Government will release the detenu the Government will not send the matter to the Advisory Board.	0.333

The similarity so computed for various documents can then be used to rank judgments in order of relevance to a query judgment.

Table 1 depicts the sample results obtained for a pair of judgments ranked as similar (ranked as 8 on a scale of 1–10) by human expert. Weight in the Table 1 represents the similarity of the sentence with the identified concept. Using the proposed approach of similarity estimation using OWA, a similarity score of 0.82 is obtained for this pair of judgments.

The following few sections present the efficacy of the proposed method using various experiments.

## Experimental Evaluation

We use Indian Supreme Court case judgments from years ranging from 1950 to 1993 for this study. These documents are used during the training phase to learn vector representations for words. Case judgments used for the experiments in this work were crawled from website http://www.judis.nic.in.

### Experimental setup

Some of the judgments documents are extremely small and may not reveal any pattern. We considered 9,372 judgments with a length of more than 10 sentences for this work. These documents are cleaned by removing the metadata information about the date, judge’s names, bench details, etc. While this information may be required for searching a particular case, it doesn’t contribute to the similarity among case judgment. Judgments contain a lot of non-text information like section and rule numbers, specific number and naming conventions used for references that include special characters that need to be preserved. Such information poses challenges in the pre-processing task and demands a domain-specific pre-processing which is important for deciding similarity. Following pre-processing steps are used in our work

(a) Preserve numbers and special characters wherever significant by removing space between word and number. Used for citation objects with numbers For Example section 23 converted to section23, clause 3(a) converted to clause 3a.
(b) Use common nomenclature for citation objects(IPC <->Indian Penal Code, Constitution of India <->Indian Constitution etc.).( Guide to Legal Citation, 2010)
(c) Perform generic linguistic pre-processing of case conversion, English stop word removal stemming and lemmatization, punctuation and number removal. Only numbers as words are removed i.e., section 23 retained but a number 456 removed.
(d) Remove Legal stop words. Some words (e.g., petitioner, petition, respondent, court, etc.) appear in almost every judgment. We construct legal stop word set forming a list of words having the highest frequency across all documents and remove these words from the documents.

The set of 9,372 judgment documents pre-processed as above is used for training in the proposed work to obtain word embedding and TF-IDF weights for words which are used for calculation of similarity. We used Gensim package Word2Vec library (Gensim, 2014) for implementation. Word2Vec function is trained on pre-processed judgment corpus. The function results in a vector representation of every word of the considered documents. We experimented with different vector dimensions for training Word2Vec. Best results were obtained for vector dimension 100 which is used for all the experiments in this work. We used Gensim TF-IDF library (Gensim, 2014) for obtaining TF-IDF weights for the words in the document collection.

**Figure 5:**
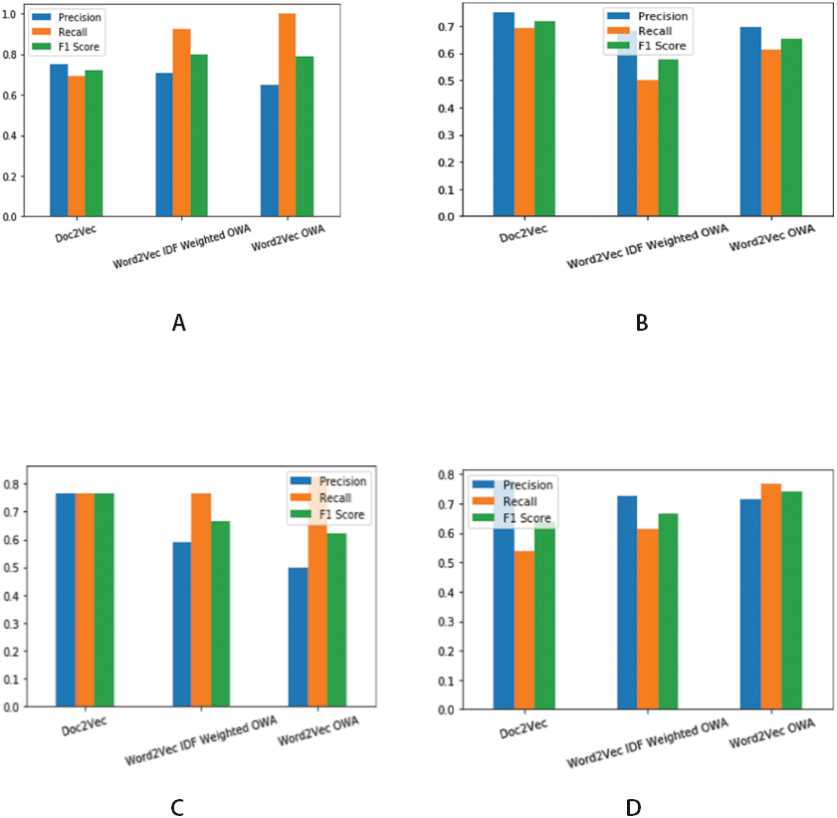
Precision, Recall and F1 score for different threshold values. (A) Precision, Recall and F1 score for Threshold value ≥ 0.4. (B) Precision, Recall and F1 score for Threshold value ≥ mean of obtained similarity values. (C) Precision, Recall and F1 score for Threshold value > 0.5 (D) Precision, Recall and F1 score for Threshold value ≥ 0.5′.

**Table 2:** Pairwise similarity estimation.

Approach	Precision	Recall	F1 Score
Word2vec using OWA	0.714	**0.769**	0.741
Word2vec idf weighted using OWA	0.706	0.920	**0.800**
Doc2vec	**0.764**	0.764	0.764

**Figure 6:**
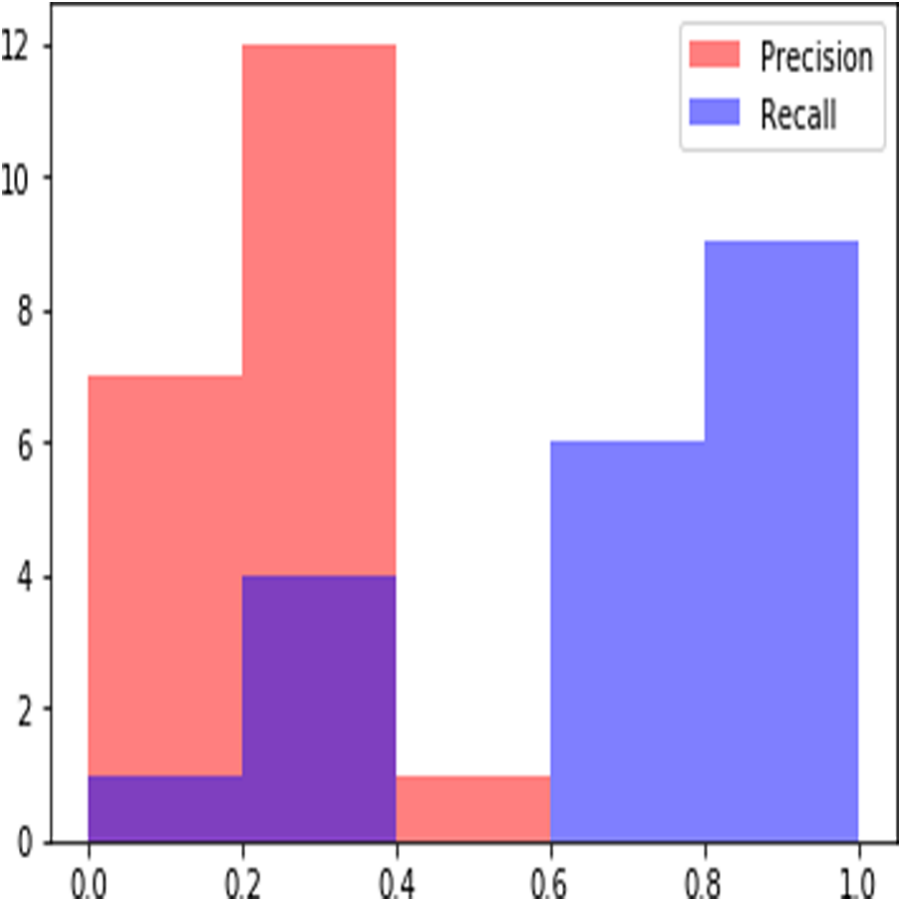
Sample Precision Recall Plot obtained at rank 20.

## Experimental Results and Discussion

Similarity estimation for legal documents facilitates two primary operations in LIR namely pairwise similarity estimation and extraction of relevant judgments from a collection of documents. The value of pairwise similarity obtained for a pair of documents, can guide a user in establishing the parallel the between two documents whereas similarity of a document with all other documents can be used for ranked retrieval in LIR. We evaluate our experiments of finding similarity among legal documents with the help of two different test approaches. We use binary classification for estimating pairwise similarity and information retrieval techniques to demonstrate the effectiveness of proposed approach in extraction of relevant documents. The following sub-sections elaborate these test approaches and the metrics used for the evaluation of the results.

1. **Pairwise similarity estimation**—We use the proposed approach of similarity estimation using OWA operator for finding similarity between a pair of case judgments. In the absence of test data for concept-wise similarity, we compare the results of our proposed approach with existing work for estimation of single similarity value for a judgment pair. We used the gold standard test dataset (Kumar et al., 2013; Mandal et al., 2017a; Mandal et al., 2017b) for this evaluation. The dataset contains relevance score given by human experts for 47 pairs of judgments of the Supreme Court of India. For every pair, experts have given similarity ratings with values between 0–10. Finding similarity among case judgments using various approaches is presented in Mandal et al. (2017a), Mandal et al. (2017b) where authors have highlighted the superiority of results obtained using the document embedding approach, Doc2Vec (Le & Mikolov, 2014). To evaluate the effectiveness of our proposed approach in identifying if a pair of judgment is similar or dissimilar, we use a simple binary classification approach. A judgment pair is labeled as similar if the obtained similarity value is greater than the chosen threshold value. We normalized expert scores to transform values in [0, 1] range and experimented classification with different threshold values. Though accuracy is the most commonly used measure of a classifier’s performance, it cannot differentiate between the number of correct labels of different classes. Hence we use precision, recall and F-measure to evaluate the effectiveness of our proposed approach. In the context of the binary classification as mentioned above, precision represents the fraction of correctly classified documents among the retrieved documents within a class and is calculated using following equation (Sokolova, Japkowicz & Szpakowicz, 2006).
  \begin{eqnarray*}Precision= \frac{TRUE POSITIVE}{TRUE POSITIVE+FALSE POSITVE} \end{eqnarray*}
  Recall is the fraction of relevant documents within a class that have been retrieved from the total number of relevant documents. Recall can be calculated using the following equation.
  \begin{eqnarray*}Recall= \frac{TRUE POSITIVE}{TRUE POSITIVE+FALSE NEGATIVE} \end{eqnarray*}
  F1 is defined as the weighted harmonic mean of the precision and recall and is computed using the following equation.
  \begin{eqnarray*}F1Score=2\ast \frac{Precision\ast Recall}{Precision+Recall} \end{eqnarray*}
  Precision, recall and F1 score together are used to evaluate classification effectiveness. Figure 5 shows the results of binary classification obtained for various threshold values. We compare our results with the existing prior work (Mandal et al., 2017a; Mandal et al., 2017b) for finding similarity among legal judgments. Thus Doc2Vec in the Fig. 5 and Table 1 represent pairwise similarity estimation using document embedding scheme reported by Mandal et al., (2017a); Mandal et al., (2017b). Table 2 presents the comparison of the results. We have included only the best case results for the experimented approaches. As described in the previous subsections, we use two vector representations namely word2vec and word2vec with idf for every word in the representative sentences for each concept. As it can be seen from Table 1, our proposed approach gives results comparable with the existing approach of document embedding. It can be seen form the results that combining idf, the inverted document frequency with the word vectors results into better F1 score of 0.8 for pairwise similarity estimation. It is also to be noted that the overall F1 score of 0.741 obtained by using only word2vec is comparable with existing approach. To evaluate the effectiveness of our proposed approach, we also performed pairwise t test of statistical significance on the F1 scores obtained for individual cases in the test dataset. The test resulted into a confidence score of 90% when compared with existing approach.
2. **Extraction of relevant judgments from a collection of documents**—We use the proposed approach of similarity estimation for extraction of relevant judgments from a collection of judgment corpus. We use ranked information retrieval technique to evaluate the effectiveness of our approach. A judgment contains references (citations) to multiple cases for justifying the validity of arguments and decisions during the proceedings of a case. These cited cases are called as precedents and are considered to have very high relevance with the citing case. For this evaluation, we construct test data as follows
  • A query set, Q is constructed by hiding all the references to precedents present in the text of the judgment. We use —Q—= 20
  • A document corpus, DC, which, along with many other judgments contains precedents i.e., the judgments cited by judgments in the query set Q. DC is used as a document base for extraction of relevant judgments. We use —DC—= 200.
  In the context of information retrieval, precision and recall are estimated differently than that of the classification approach and can be explained using following equations:
  \begin{eqnarray*}Precision& = \frac{{|}\{Retrieved Document\}\cap \{Relevant Document\}{|}}{{|} \left\{ RetrievedDocuments \right\} {|}} \end{eqnarray*}
  \begin{eqnarray*}Recall& = \frac{{|}\{Retrieved Document\}\cap \{Relevant Document\}{|}}{{|} \left\{ RelevantDocuments \right\} {|}} . \end{eqnarray*}
  In a ranked information retrieval system, precision and recall values calculated at given cut-off k, i.e., precision@k and recall@k are used as evaluation metrics (Manning, Raghavan & Schütze, 2010). Precision recall values can be plotted to obtain Precision recall curves which provide visual exploration of retrieved results at different levels of precision and recall. Interpolating precision to next higher recall is a common practice to obtain a smoother precision recall curve (Manning, Raghavan & Schütze, 2010). Figure 6 shows sample precision recall curves obtained for a query. When cut off is taken at R, the known number of relevant documents in the corpus, it is called as R-precision which is used extensively for evaluation of results in ranked retrieval systems. We use precision@k, R-precision and recall@k for the evaluation of the results of our proposed approach. Results obtained for different values of k are summarized in Table 3. We compare the results with previous work on the extraction of relevant judgments of the supreme court of India (Mandal et al., 2017a; Mandal et al., 2017b). In this work best performance of retrieval is obtained by considering only the citation context, i.e., the paragraph around the case citation in a judgment and then applying inverted document frequency, IDF for estimation of similarity. As it can be seen from the results presented in Table 2, our proposed approach clearly outperforms the existing work. We obtain the best result of 0.318 for R-precision which highlights the effectiveness of the proposed result for ranked retrieval of judgments. The proposed approach also results in higher values of recall for a smaller cut of value, *k* = 50 ascertaining its efficacy in retrieving relevant judgments within a document collection.

## Conclusions

Establishing relevance among legal documents is a very complex task and demands specialized approaches to similarity estimation. In this paper, we presented a novel approach of extracting prominent concepts from the document for finding the similarity among legal judgments. We presented legal document similarity estimation as a multi-criteria decision-making problem which we solved using aggregation operator OWA. In addition to the improvement in the results, the proposed approach provides multiple levels of similarities which facilitates visualization and can be useful for deeper insights into the notion of relevance among court judgments. The presented approach is entirely data-driven as the concepts to be matched are extracted intrinsically and there is no need for the user to formulate a query. The proposed approach also extracts sentences specific to every concept and set of these sentences can be used as a condensed representation for the judgment document. The proposed approach used common nouns to identify basic concept words. In future, we would like to use more sophisticated methods like named entities and entity co-references for identification of concepts. Community detection algorithms based on centrality and between-ness can be explored for the identification of prominent communities. We would like to explore the possibility of introducing the concept weighting scheme based on the importance of a concept in various sub-domains of law for a deeper understanding of relevance.

**Table 3:** Extraction of relevant Judgments: ranked Information retrieval.

	Proposed approach						Existing work		
Method used	Precision @10	Precision @r	Recall @20	Recall @50	Recall @100	Method Used	Precision @10	Recall @ 100
Word2vec	0.205	**0.243**	0.638	**0.805**	**0.916**	IDF, citation context	0.236	0.781
							Parsimonious language model, citationcontext	0.237	0.771
Word2vec with idf weight	**0.225**	**0.318**	0.673	**0.847**		**0.923**	Citation context	0.221	0.749
							Dirichlet Prior Smoothing	0.218	0.681

## Additional Information and Declarations

### Data Availability

The following information was supplied regarding data availability: The data is available at Figshare: wagh, rupali (2019): Document corpus of court case judgments. figshare. Dataset. https://doi.org/10.6084/m9.figshare.8063186.v2.
